# Memory–Non-Linearity Trade-Off in Distance-Based Delay Networks

**DOI:** 10.3390/biomimetics9120755

**Published:** 2024-12-11

**Authors:** Stefan Iacob, Joni Dambre

**Affiliations:** IDLab-AIRO, Faculty of Engineering and Architecture, Ghent University, 9052 Ghent, Belgium

**Keywords:** information processing capacity, memory capacity, memory–non-linearity trade-off, reservoir computing, distance-based delay networks, echo state networks

## Abstract

The performance of echo state networks (ESNs) in temporal pattern learning tasks depends both on their memory capacity (MC) and their non-linear processing. It has been shown that linear memory capacity is maximized when ESN neurons have linear activation, and that a trade-off between non-linearity and linear memory capacity is required for temporal pattern learning tasks. The more recent distance-based delay networks (DDNs) have shown improved memory capacity over ESNs in several benchmark temporal pattern learning tasks. However, it has not thus far been studied whether this increased memory capacity comes at the cost of reduced non-linear processing. In this paper, we advance the hypothesis that DDNs in fact achieve a better trade-off between linear MC and non-linearity than ESNs, by showing that DDNs can have strong non-linearity with large memory spans. We tested this hypothesis using the NARMA-30 task and the bitwise delayed XOR task, two commonly used reservoir benchmark tasks that require a high degree of both non-linearity and memory.

## 1. Introduction

Temporally complex machine learning tasks often require a combination of memory and non-linear processing. As such, using a computational substrate that facilitates a combination of non-linear computing and a large short-term memory span is beneficial for processing spatiotemporal information. However, in dynamical systems, there is a trade-off relation between linear memory and non-linear dynamics, since non-linear computations degrade memory [1,2]. This poses a problem for tasks that require both.

Reservoir computing (RC) is a brain-inspired machine learning paradigm based on harnessing the intrinsic temporal processing properties of dynamical systems. These dynamical systems, referred to as reservoirs, serve as temporal kernels [3] that map a low-dimensional input signal to a high-dimensional projection, which is then fed to a simple linear readout model. This increase in dimensionality usually increases the linear separability of the data. Traditionally, this involved (software-simulated) recurrent neural networks (RNNs) as reservoirs. More recently, the field of RC has been mainly focused on physical reservoir computing, where physical hardware systems such as photonic, electronic, or memristive hardware are used as reservoirs [4,5,6]. These physical substrates have the advantage of being fast, since they do not need to be simulated on traditional hardware, and energy-efficient, with many physical reservoirs being mostly passive [7]. This speed and efficiency offers the possibility of real-time processing with applications in speech recognition [8,9], forecasting and signal generation [10,11], and robot control [12].

However, besides physical reservoir computing, software-simulated reservoir computing is still used as a model system for physical RC, and as a test-bed for novel brain-inspired mechanisms (e.g., [13,14,15]). Some of the earliest software-simulated RC frameworks were echo state networks (ESNs), introduced by [16] as an alternative to optimizing recurrent weights using back-propagation through time (BPTT). ESNs consist of a fixed RNN reservoir with randomly initialized weights and a linear regression readout layer. The neurons are simple hyperbolic tangent or sigmoid units.

ESNs have good performance in tasks with temporal dependencies, while requiring no RNN weight training. However, because ESN reservoirs are randomly initialized, they can be seen as a sample from a distribution. Although RNN weights are fixed, these distribution parameters need to be optimized as hyperparameters for specific tasks.

In the RNNs used as ESN reservoirs, non-linear processing is provided by non-linear neuron activation functions. Furthermore, the maintains part of the input information through the use of recurrent connections and leak parameters, providing the system with short-term memory. Many temporal machine learning problems require a combination of linear memory and non-linear processing, with the span of this short-term memory varying per task, and even within the same task. ESNs are highly flexible in this regard. Due to a high-dimensional and temporally dependent reservoir state, they provide information from a wide temporal range. However, refs. [1,2] show that, to increase ESN memory without increasing the reservoir size, reservoirs have to operate in a more linear regime. In dynamical systems, memory, especially for longer time spans, is in direct competition with the non-linear processing capacity. Intuitively, the amount of information about the input signal that can be retained in the reservoir is diminished by altering that information with non-linear transformations. The only way to increase both memory and non-linear processing is by increasing the reservoir size (i.e., increasing the number of neurons). This memory–non-linearity trade-off puts ESNs at a disadvantage for tasks that require recombining information non-linearly across a large memory span.

A recently introduced class of ESNs with brain-inspired adaptations, called distance-based delay networks (DDNs) [17], achieve a higher performance for several highly non-linear temporal benchmark tasks. In [14], we showed that the variable inter-neuron delays introduced in DDNs can be optimized to match network timescales to task requirements. Various (modeled) physical reservoir computing approaches commonly employ delay connections [18,19,20,21], but are usually limited to individually engineered feedback connections rather than task-optimized distributions of delays. In contrast, in DDNs, inter-neuron delays are proportional to the inter-neuron distance, inspired by the properties of biological axons. The axonal signal propagation speed depends on the myelination and axon diameter [22], but simplifying these two factors, an axon applies a delay proportional to its length. As such, DDNs use the inter-neuron distance as a determining factor for the delay size.

The use of varying axonal propagation delays has been shown to be essential for several neural pathways: among others, for sensory processing, coincidence detection, and motor control [23,24,25]. Moreover, a large variety of axonal delay ranges can be observed in most mammals [26], suggesting an evolutionary advantage of maintaining a variety of delays. Not all inter-neuron delays are axonal, with some being caused by integration in dendrites, somata, and synapses, which also include non-linear computations. In contrast, axonal delays are linear, transmitting action potentials without any filtering.

In [27], we showed an increase in linear memory capacity without increasing the reservoir size and without swapping non-linear neurons with linear neurons. It was hypothesized that the improved DDN performance was the result of an improved memory–non-linearity trade-off. It was also shown that DDNs indeed have a higher linear memory capacity concentrated over larger time spans. However, it was not systematically tested whether linear memory capacity is not increased at the cost of non-linear processing. In this paper, we confirm that DDNs indeed achieve a better balance between linear memory and non-linear processing over larger time spans. DDNs offer a more flexible tuning of memory and non-linear processing, leading to a better balance between the two. The novel contribution presented here is an empirical proof of an improved memory–non-linearity trade-off when using tuneable inter-neuron delays.

We first show that randomly introducing distributed inter-neuron delays in unoptimized networks already has a measurable effect on the memory–non-linearity trade-off.

Using the information processing capacity (IPC) framework introduced by [1], we quantified the computational properties of the reservoir, the computational requirements of tasks, and the overlap between the two. We show that task performance is correlated with the task overlap. We show that DDNs optimized for the NARMA-30 task (a commonly used RC benchmark task) indeed achieve an increase in non-linear processing with larger memory spans, without a decrease in task-relevant linear memory, which better aligns with the task requirements.

Lastly, we optimized DDNs and ESNs for the delayed XOR task [28], a highly non-linear task with easily interpretable short-term memory span requirements. The delayed XOR task requires non-linearity at increasingly large lags, depending on the task parameter, which is challenging for conventional ESNs. We show that the performance of ESNs decayed faster than that of DDNs as we increased the delay in this task.

## 2. Background

ESNs were initially introduced as a computationally efficient alternative to traditional RNNs trained using BPTT. Instead of BPTT, ESNs utilize a fixed recurrent ‘reservoir’ comprised of (usually leaky) sigmoid or hyperbolic tangent neurons. Only a linear readout layer undergoes training. Conventional ESN reservoirs are input-driven dynamical systems described by the following equation.

(1) $$\begin{array}{cc} x(n+1) & =\left(1-a\right)x\left(n\right)+a\cdot f(W_{\mathrm{res}}x\left(n\right)+b_{\mathrm{res}}+W_{\mathrm{in}}v\left(n\right)) \end{array}$$

In this context, the variables x(n), *a*, $W_{\mathrm{res}}$, $W_{\mathrm{in}}$, $b_{\mathrm{res}}$, *f*, and v(n) denote the network state at timestep *n*, the decay rate, the weight matrix for the (recurrent) reservoir, the weight matrix for inputs, the bias weights of the reservoir, the activation function, and the input at timestep *n*, respectively.

Balancing memory with non-linear computation is a fundamental problem in dynamical systems and RNNs that are used as reservoirs, and has received much attention in recent publications [1,2,29,30]. Alongside the aforementioned DDNs, several ESN adaptations are claimed to improve this memory–non-linearity trade-off, which usually means increasing the memory of a system without degrading its non-linear processing power, or vice versa.

Indeed, ref. [2] show that non-linear activation functions work best in tasks that require strong non-linear transformations with a short memory, and tasks requiring a long memory with weak non-linear transformations are best solved using linear ESNs. However, in the case of tasks that require both memory and strong non-linearity, mixture reservoirs, which use a combination of linear and non-linear neurons, outperform both linear and non-linear conventional ESNs [2]. Similarly to DDNs, mixture reservoirs include some degree of linear processing; however, DDNs do so without reducing the number of non-linear nodes, by offloading the linearity to inter-neuron delays. In the case of physical reservoirs, inter-node delay is computationally free, as it is inherent to the system.

Another approach, called an edge of stability ESN (ES^2^N), is shown in [29]. Here, the proximity of the reservoir to chaotic behavior can be manipulated by hyperparameter tuning. Specifically, instead of having a single leak parameter for the entire network, individual leak parameters are optimized per neuron by scaling the leak rate with an orthogonal matrix. The resulting networks are shown to perform better at the same tasks used in [2], which were designed to require strong non-linear transformations with large short-term memory spans.

Although both models were validated on tasks that require high degrees of non-linearity and large memory spans, it is difficult to formally quantify whether the trade-off is really improved. The balance between linear memory and the non-linear IPC is formalized in [1]. The IPC measures the capacity of a dynamical system to reconstruct a set of the non-linear basis functions of its past inputs. As such, capacities can be measured for different degrees of non-linearity and across different memory spans. However, it has been proven that the total IPC, which is the sum of all possible IPC components, is always equal to the number of observed variables, if the dynamical system has the fading memory property and linearly independent state variables. As such, it is impossible to increase the theoretical total IPC for reservoirs with the same number of neurons. The memory–non-linearity trade-off, as originally defined, cannot be improved, as increasing linear memory in a system will always decrease the remaining IPC. Nevertheless, improvements in tasks that require both are presented in [2], indicating that in practice, the amount of the ‘usable’ IPC (i.e., task-relevant IPC) can be improved. In addition, we argue that a better balance between memory and non-linearity does not require a higher total IPC, but simply requires a high non-linear IPC at large lags (i.e., applying non-linear transformations on memorized inputs). Tasks that require non-linear transformations on inputs from *n* steps ago require a high non-linear memory capacity at a lag of *n*, and not necessarily for all lags up to *n*. Therefore, even though the theoretical total IPC is fixed, here we define an improved memory–non-linearity trade-off as having a better balance between the two at regions in the IPC landscape that coincide with task information processing requirements. In theory, this can be achieved by decreasing the IPC in ‘useless’ regions (i.e., for degrees of non-linearity and lags not required by the task). For linear memory capacity, this has already been shown for DDNs in [27]. In this paper, we show that the ability to focus memory at desired lags allows for a more useful IPC, which leads to improved task performance.

## 3. Methods

### 3.1. DDNs

DDNs were introduced in [17] as an extension to conventional ESNs. They were inspired by the computational properties of biological axons. Instead of instantly transmitting a signal from one neuron to another, axons apply a finite signal propagation delay, depending on various factors such as the length and diameter [26]. Similarly, DDNs simulate this phenomenon in a simplified way, by introducing distance-dependent signal delays. Neurons are defined with a corresponding spatial location, and the Euclidean distance between each neuron pair dictates the amount of delay applied to connection $w_{i,j}$. DDNs are formalized as follows.

(2) $$\begin{array}{cc} x(n) & =\left(1-\alpha \right)x\left(n-1\right)+\alpha f\left(\sum_{d=0}^{D_{\max}}\left(W_{D=d}^{\mathrm{res}}x\left(n-d\right)+W_{D=d}^{\mathrm{in}}v\left(n-d\right)\right)+b_{\mathrm{res}}\right) \end{array}$$

Here, x(n) is the reservoir state at time *n*, α is the decay parameter (also referred to as the leak rate), f(·) is the activation function (in this case, the hyperbolic tangent), v(n) is the task input at time *n*, and $b_{\mathrm{res}}$ is the vector of reservoir bias weights. Instead of a single full weight matrix, as in Equation (1), we now sum over multiple sparse weight matrices that only contain the weights corresponding to connections of a (discretized) distance *d* between any two neurons, with all other elements in the matrix set to 0. The elements of this delay-specific weight matrix $W_{D=d}$ are defined by

(3) $$\begin{array}{cc} W_{i,j,D=d} & =\delta_{d,D_{i,j}}\cdot W_{i,j} \end{array}$$

where $\delta_{\cdot ,\cdot }$ refers to the Kronecker delta operator.

### 3.2. Hyperparameter Optimization

As mentioned, both ESNs and DDNs are randomly initialized networks, which can be seen as samples from a hyperparameter-defined distribution. Although conventional ESNs are usually defined as a ‘single, homogeneous reservoir’ (i.e., all neurons are sampled from the same hyperparameter distribution), in [17] we compared both single-reservoir and multi-reservoir ESNs to equivalent DDN counterparts. In the case of multi-reservoir ESNs, this means that neurons are sampled from a multimodal hyperparameter distribution. We showed that multi-reservoir systems outperform single reservoirs, both in the case of ESNs and DDNs. As such, in this work, both ESN and DDN systems were optimized as a multi-reservoir system, where hyperparameters were defined for multiple sub-reservoirs (referred to as clusters), and connection-related hyperparameters were defined between and within each cluster. ESN hyperparameters were the means and standard deviations of the reservoir weights $W_{\mathrm{res}}$ defined per cluster pair, the leak parameters α, and the mean and standard deviation of the bias weights $b_{\mathrm{res}}$ defined per cluster. In addition to the standard ESN hyperparameters, DDNs also specified a distribution of neuron spatial coordinates. These were sampled from a multi-modal Gaussian distribution, whose parameters were optimized along with the standard ESN hyperparameters.

To optimize these hyperparameters for specific tasks, we made use of the covariance matrix adaptation evolutionary strategy (CMA-ES) [31], which is an evolutionary optimization technique suitable for highly irregular error landscapes. The CMA-ES is commonly used for RC hyperparameter tuning [32,33]. We sampled 20 candidate hyperparameter sets for each generation. From each candidate, we randomly sampled five reservoirs. We optimized DDNs and ESNs for the delayed bitwise XOR task and the NARMA-30 task, which are further explained in Section 3.6. We trained and validated each reservoir, and used the normalized root mean squared error (NRMSE) on the validation set, averaged over the five reservoirs as a fitness measure for the candidates. Each model type (i.e., the ESN and DDN) was optimized over 200 generations. In the remainder of this paper, unless otherwise specified, we analyze the properties of reservoirs sampled from the best hyperparameter candidate throughout the evolution.

### 3.3. Memory Capacity

A way to quantify the amount of short-term memory a dynamical system has is the linear memory capacity (MC), first introduced in [34]. To compute the linear MC, a reservoir is driven with a random, uniformly distributed input signal. The reservoir activity is used to train a readout layer that reproduces the input from *l* timesteps ago. We refer to *l* as the lag parameter. The lag-specific MC is defined by how well the reproduction correlates with the input from *l* timesteps ago, whereas the total linear MC is simply the sum over all possible lags. The total linear MC and lag-specific MC are formalized as, respectively,

(4) $$\begin{array}{cc} MC_{tot} & =\sum_{l=1}^{\infty }MC_{l} \end{array}$$

(5) $$\begin{array}{cc} MC_{l} & =r{\left(u\left(n-l\right),{\hat{u}}_{l}\left(n\right)\right)}^{2} \end{array}$$

In this equation, r(·,·) is the Pearson correlation coefficient, u(n) is the input to the reservoir at time *n*, and ${\hat{u}}_{l}\left(n\right)$ is the readout estimate for u(n−l). Since the estimator used to reproduce the inputs is a simple linear regression, MC is reservoir-specific, not readout-specific, and can be used as a measure to characterize the memory properties of a network. Lag-specific MC profiles show how far in the past an RNN retains most input information. For conventional ESNs, $MC_{l}$ monotonically decreases as *l* is increased, meaning that ESNs will always have less information about temporally distant inputs, which is related to the ESN’s fading memory property. However, in [27] it was shown that DDNs are able to focus MC at specific lags, without needing a high MC at lower lags.

### 3.4. Information Processing Capacity

Linear memory capacity was later generalized by [1] to the IPC, to include non-linear processing. Instead of reproducing the delayed input, a non-linear function of the input is estimated by the readout layer. As long as the set of non-linear functions form an orthogonal basis for the used i.i.d. input domain, the individual capacities corresponding to each basis function should be independent, and thus measure separate computing properties of the network. The information processing capacity was originally only defined for discrete time systems such as ESNs, although this was later extended to time-continuous systems such as spiking neural networks [30].

In this work, we analyzed the IPC for systems driven with an input that was uniformly distributed over the interval [−1,1]. For this input domain, an orthogonal basis was formed by the products of all possible combinations of normalized Legendre polynomials. The IPC was then given by measuring all possible capacities, with each individual capacity being defined by the lag parameter *l* (meaning that we reproduced a function with input from *l* steps ago) and the combination of polynomials $\left\{d_{i}\right\}$. This capacity is defined as

(6) $$\begin{array}{cc} C_{l}^{\left\{d_{i}\right\}} & =r{\left(z_{l}^{\left\{d_{i}\right\}},{\hat{z}}_{l}^{\left\{d_{i}\right\}}\left(n\right)\right)}^{2} \end{array}$$

where *r* is Pearson’s correlation, $\left\{d_{i}\right\}$ is the set of polynomial degrees used for this capacity, $z_{l}^{\left\{d_{i}\right\}}$ is the function to be reproduced, and ${\hat{z}}_{l}^{\left\{d_{i}\right\}}\left(n\right)$ is the readout reproduction based on the reservoir activity at time *n*. The product of polynomials is defined as

(7) $$\begin{array}{c} z_{l}^{\left\{d_{i}\right\}}=\prod_{i}P_{d_{i}}\left(u\left(n-l-i\right)\right) \end{array}$$

where $P_{d}\left(\cdot \right)$ is the normalized Legendre polynomial of degree *d* and u(n−l) is the input of *l* steps ago. As such, for a lag of *l*, the function to be reproduced can be any combination of polynomials of inputs from at least *l* steps ago. The set $\left\{d_{i}\right\}$ can contain any combination of polynomials with degrees larger than zero. With these conditions, each capacity is guaranteed to capture the independent computational properties of the system. Because we wanted to characterize the trade-off between memory and non-linearity, we ordered capacities based on their (maximum) degree (i.e., the largest *d* in each set, $\left\{d_{i}\right\}$) and the lag, *l*. As such, linear memory was defined by the IPC of degree 1 and non-linear processing by larger degrees. As we computed the IPC for higher maximum degrees, we saw a combinatorial explosion of capacities, as the amount of combinations possible for set $\left\{d_{i}\right\}$ increased. Similarly to the approach in [1], we therefore limited the amount of measured capacities, not only by the maximum degree, but also by a maximum window size, *w*, such that i≤w, where *i* refers to the amount of delay steps above the lag (which is the smallest delay in the set of polynomials). This effectively limited the temporal distance between the latest and the earliest component of the capacity, thus reducing the IPC search space. Next, the total maximum number of polynomials, *v*, was limited, such that the size of the set $|\{d_{i}\}|\leq v$, where $\left|\{d_{i}\}\right|$ refers to the size of the set of IPC polynomials. By limiting the set size of polynomials, we again reduced the number of capacities that needed to be computed. As such, the maximum window size and maximum number of polynomials shape the search space of the IPC, and should be informed by domain knowledge of the task to which the reservoir system is applied (further discussed in Section 3.5).

The total theoretical IPC is the sum of all capacities for lags 0 to infinity, degrees 1 to infinity, and all possible combinations of polynomials. It was shown in [1] that the total IPC is always upper bounded by the amount of observed variables if these variables are linearly independent and the dynamical system has fading memory. However, in practice, we only measured capacities for a finite number of lags and degrees, and we only counted capacities above a minimum, statistically relevant threshold and within the previously mentioned maximum window size and maximum number of polynomials, *w* and *v*, respectively. An IPC profile that is too thinly spread out over a large region (i.e., over too many lags and degrees of non-linearity) is generally not useful in tasks that require strong non-linearity and memory at specific points in time. However, more ‘focused’ capacities do not necessarily mean that they are useful, as usefulness is purely task-dependent. We therefore needed to define the region of useful capacities by quantifying how relevant each capacity $C_{l}^{\left\{d_{i}\right\}}$ was for a specific task.

### 3.5. Task Capacities

To obtain insight into where the ‘useful’ regions of the IPC were, we used the notion of task capacity. To compute task capacity, instead of using a dynamical system to reproduce the basis functions of past inputs, we measured the correlation with the outputs of the task system itself and the basis functions of its past inputs. As such, for first-degree basis functions, we had an indication of points at which lags in linear memory were needed. For higher degree basis functions, peaks in the task capacity showed where in time non-linear transformations were needed. By comparing the task capacity with the optimized ESN and DDN IPC, we can have a better understanding of how well memory and non-linearity are balanced in a task-specific context. Thus, we here define task capacity similarly to Equation (6) as

(8) $$\begin{array}{cc} TC_{l}^{\left\{d_{i}\right\}} & =r{\left(z_{l}^{\left\{d_{i}\right\}},y\left(n\right)\right)}^{2} \end{array}$$

where y(n) is simply the state of the task system (i.e., the system to be predicted by the reservoir) at time *n*, and $z_{l}^{\left\{d_{i}\right\}}$ is the same as in Equation (7). Large task capacities indicate a large correlation between the current system state and the function, *z*, of its past input, meaning that *z* is a task-relevant (useful) IPC component. As such, measuring task capacity allowed us to compare a reservoir’s IPC profile with the task profile. Moreover, to quantify the portion of the task-relevant IPC, we defined an overlap measure as an inner product between the task capacity profile and the equivalent reservoir capacities, formalized as

(9) $$\begin{array}{c} o=\sum_{\left\{d_{i}\right\}}\sum_{l}C_{l}^{\left\{d_{i}\right\}}TC_{l}^{\left\{d_{i}\right\}} \end{array}$$

This is simply a weighted sum of the IPC, where capacities have a higher weight if the corresponding task capacity is high.

If the task IPC overlap is indeed a good predictor for task performance, the two should strongly correlate. Following this, for tasks where DDNs outperform ESNs, DDNs should achieve a better task overlap than ESNs. In Section 4, we show that this is true for both unoptimized and optimized reservoirs.

### 3.6. Tasks

#### 3.6.1. NARMA

We analyzed multi-cluster ESNs and DDNs, which were optimized for the nonlinear autoregressive moving average (NARMA) system approximation task [35]. This task is commonly used as a benchmark for RC systems, and requires both non-linear processing and time-specific memory. The NARMA system is defined by the following equation.

(10) $$\begin{array}{c} y\left(t+1\right)=c_{1}\cdot y\left(t\right)+c_{2}\cdot y\left(t\right)\sum_{i=0}^{p}y\left(t-i\right)+c_{3}\cdot u\left(t-p\right)u\left(t\right)+c_{4} \end{array}$$

Here, $c_{1}$ to $c_{4}$ are task parameters, and *p* is the NARMA order. y(t) represents the state of the NARMA system at time *t*, while u(t) is the system input at time *t*. u(t) is uniformly sampled between 0 and 0.5. Given the serially presented previous inputs of the NARMA system, the task objective is to forecast the subsequent state (so-called one step ahead prediction). The reservoirs we used were optimized for NARMA-30, with p=29, $c_{1}=0.2$, $c_{2}=0.04$, $c_{3}=1.5$, and $c_{4}=0.001$, using a training set of 8000 samples and a validation set of 4000 samples.

An important practical benefit of using the NARMA benchmark in our experiments was that the task capacity could be easily computed and was upper bounded to 1 if the inputs were rescaled to the input distribution corresponding to the IPC polynomials (i.e., in this case, a uniform distribution over the interval [−1,1]).

#### 3.6.2. Delayed XOR

We optimized ESNs and DDNs for the delayed XOR task. This highly non-linear Boolean task was first introduced in [28], and is commonly used as a benchmark for (physical) RC systems [36,37,38]. For this task, a random bitstream is presented to the reservoir. The task objective is to perform the bitwise XOR operation y(t)=u(t)⊕u(t−k) between the current input, u(t), and the input of *k* timesteps ago, u(t−k). During hyperparameter optimization, both the training bitstream and the validation bitstream consisted of 2000 samples each. During evolution, the NRMSE of the readout predictions was used as the CMA-ES fitness measure. However, in Section 4, we report the bit error rate (BER), defined as the number of misclassified bits over the total number of bits in the presented bitstream.

Besides being highly non-linear, the delayed XOR task requires maintaining input information for exactly *k* timesteps. For higher values of *k*, the IPC is needed at larger lags. Thus, good performance on a delayed XOR with a large *k* implies a better memory and non-linearity trade-off. In this paper, we consider the *k*-delay bitwise XOR task for delays 1 through 19.

## 4. Results

In this section, we analyze and discuss the effect of delays on the memory–non-linearity trade-off by performing a range of experiments. Because individual IPC components each measure independent computational properties, and only some computations are useful for a task, we made use of the task overlap metric defined in Section 3.5. We first measured the task capacities of the NARMA-30 task (shown in the bottom part of Figure 5 and on the right hand side of Figure 6). Because we knew the task equation, we could constrain the search space accordingly, namely to a maximum degree of two, a maximum window size of 30, and a maximum lag of around 30 timesteps. The task capacity determined which capacities we considered useful, and could therefore be used to drastically constrain the search space for the reservoir IPC. Because we knew that the lags and basis functions for which the task capacity was zero were not relevant, we could exclusively measure the reservoir capacities that had a corresponding non-zero task capacity.

Next, we measured the IPC of unoptimized DDNs and their identical ESN counterparts. With this comparison, we provided a baseline quantification of the effects of distributed delays on the IPC and NARMA-30 task performance. Here, the presence or absence of delays could be isolated as the only independent variable, since all other network parameters were identical for each ESN and DDN pair.

We then present a comparison between ESNs and DDNs in terms of task performance in the NARMA system approximation task and the non-linear memory capacity of the optimized systems. For this experiment, the ESN and DDN models required separate hyperparameter optimizations, resulting in different network structures. The co-evolution of delays along with other network hyperparameters led to different optimal network structures than those of optimized ESNs. However, by comparing evolved DDNs and ESNs, we show improved optimization flexibility for DDNs.

Lastly, we provide another empirical proof of the superior memory–non-linearity trade-off of DDNs compared to ESNs by training and evaluating them on the delayed-XOR tasks.

### 4.1. Unoptimized Networks

We randomly initialized (single-cluster) DDNs of 100 neurons, with normally distributed neuron locations centered around the input neuron, with a standard deviation corresponding to two delay steps in both the *x*- and *y*-axis and a correlation of zero between the x and y coordinates. The weights of these networks were uniformly distributed, centered around zero, with half of the weights set to zero (i.e., a connectivity parameter of 0.5). The bias weights were distributed uniformly between zero and one. We repeated the initialization with a varying spectral radius between 0.1 and 2 and leak parameter between 0.3 and 1 over five steps each. For each parameter combination, we repeated the initialization twice and measured the IPC for all capacities up to a degree of three, a lag of 60, a maximum window size of 30, and a maximum of two polynomials. To this end, we first generated the network activity with the same input set of 6000 samples distributed uniformly between minus one and one, and then measured the capacities using this network activity. The purpose of these measurements was to illustrate the difference in the average IPC profile between ESNs and DDNs; hence, the granularity of the parameter space was limited in the interest of computation time.

**Figure 1:**
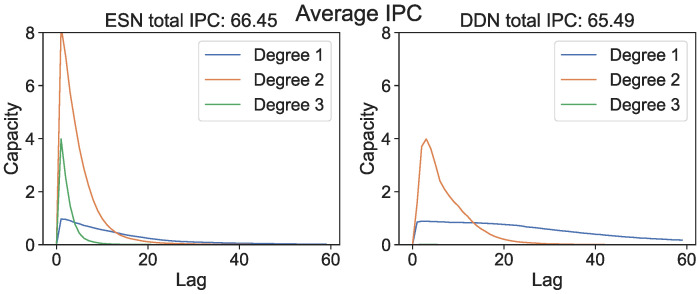
Average IPC profile for randomly initialized unoptimized ESNs and DDNs.

In Figure 1, we show the IPC (i.e., capacities plotted against the lag for all measured degrees) averaged over all network initializations and all parameter settings. Although ESNs have a lower average total measured IPC compared to DDNs, we see that (at least for unoptimized networks) DDNs are more linear, with the IPC profiles spanning larger lags, whereas unoptimized ESN IPC profiles are concentrated in more non-linear regions, but reach smaller lags.

**Figure 2:**
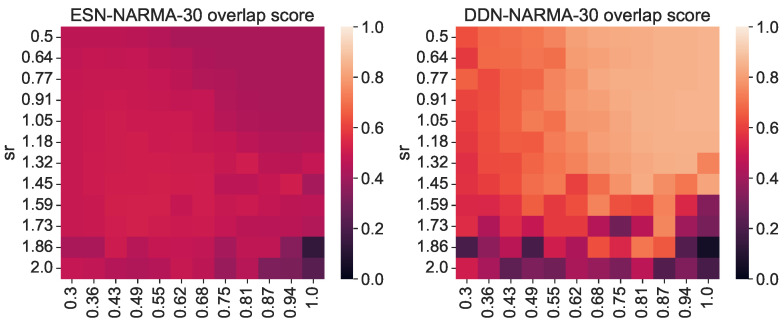
Task–reservoir IPC overlap for various random network initializations. The right-hand side shows the overlap scores of DDNs initialized with random, normally distributed neuron locations. The left-hand side shows the overlap of equivalent ESNs. All scores are averaged over 3 random initializations with the same leak rate and spectral radius.

As stated earlier, the total IPC should in theory always be equal to the number of observed variables; hence, the difference in the total measured IPC must either lie in unexplored regions (e.g., basis functions with more polynomials) or be spread out over many statistically insignificant (hence sub-threshold) capacities. As such, we must return to the concept of useful capacities to be able to explain the performance difference between DDNs and ESNs. To this end, we repeated the previous experiment, but instead of measuring the IPC over the full search space, we measured the region-specific IPC (i.e., we only measured those capacities that had a corresponding non-zero task capacity). Limiting the number of measured capacities reduced the computational load, so for this experiment, we increased the granularity for the spectral radius and leak rate space to 12 steps, and we repeated each network instantiation three times. Using the region-specific IPC, we then computed the task overlap as the inner product between the vector of task capacities and the reservoir IPC, as shown in Equation (9). For each network, we repeated the experiment with its ESN counterpart, obtained by setting all delays to zero. As such, we compared ESNs and DDNs which only differed in terms of delay, while keeping all other network parameters identical (i.e., the weights, input weights, biases, connectivity, and leak rate). In Figure 2, we present the resulting task overlap for each combination of the leak rate and spectral radius. We see that the overlap is higher for DDNs for most parameter combinations, and also reaches a higher maximum overlap.

**Figure 3:**
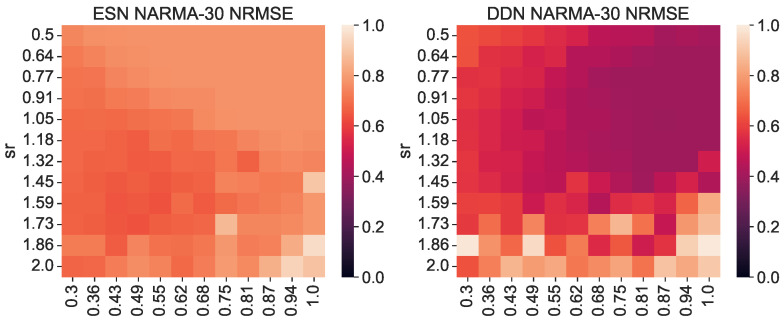
NARMA-30 NRMSE for various random network initializations. The right-hand side shows the performances of DDNs initialized with random, normally distributed neuron locations. The left-hand side shows the performance of equivalent ESNs. All scores are averaged over 3 random initializations with the same leak rate and spectral radius.

**Figure 4:**
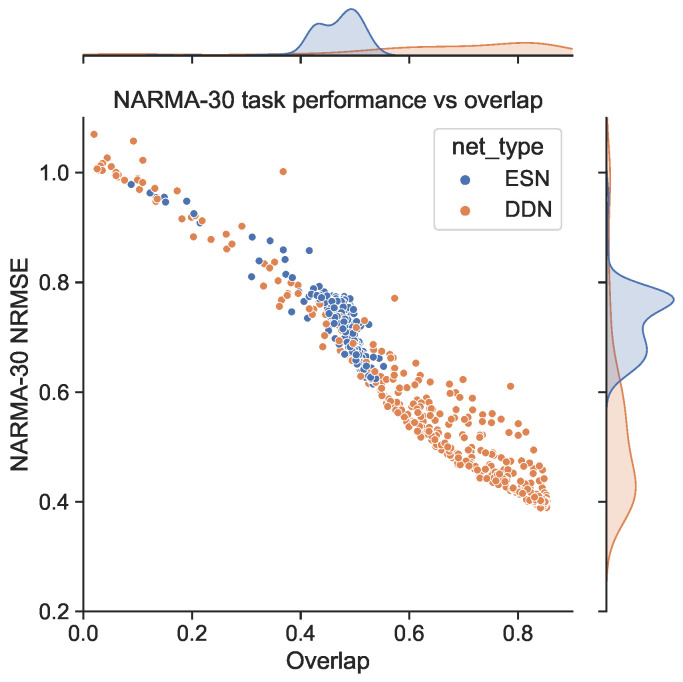
Scatter plot of the task IPC overlap against task performance for the ESNs and DDNs from Figure 2 and Figure 3. Each dot corresponds to a single initialization and network simulation of an ESN or DDN. All DDNs have their equivalent ESN, which is identical in all aspects except inter-neuron delays.

We generated a NARMA-30 training set of 4000 samples to train the readout layers. Then, using the same random inputs and corresponding network activity used to measure the IPC before, we generated NARMA-30 outputs and evaluated the network performance on the NARMA-30 task (again, the same set for each evaluation). Because the input for measuring the IPC is distributed uniformly between −1 and 1, when generating the network activity and NARMA-30 outputs, we rescaled the input to the range of 0, 0.5 required for the NARMA tasks. In Figure 3, we show the corresponding NARMA-30 NRMSE. Note that these are the exact same network initializations as in Figure 2. Again, we see that DDNs achieve a lower NRMSE, confirming findings in earlier work. More interestingly, we observe a (negative) correlation between areas of high task overlap in Figure 2 and areas of low task error in Figure 3. This correlation is particularly pronounced for reservoirs with a larger spectral radius. Moreover, in Figure 4, we see that these two metrics (task performance and overlap) are strongly correlated, with a higher task overlap being associated with a lower task NRMSE. Note that the ESN initializations are more clustered than the DDNs, whereas DDNs are more spread out, but reach a much lower NRMSE. Although we observe this strong correlation for both network types, we see that DDNs especially produce some instances that seem to behave as outliers.

### 4.2. NARMA

**Figure 5:**
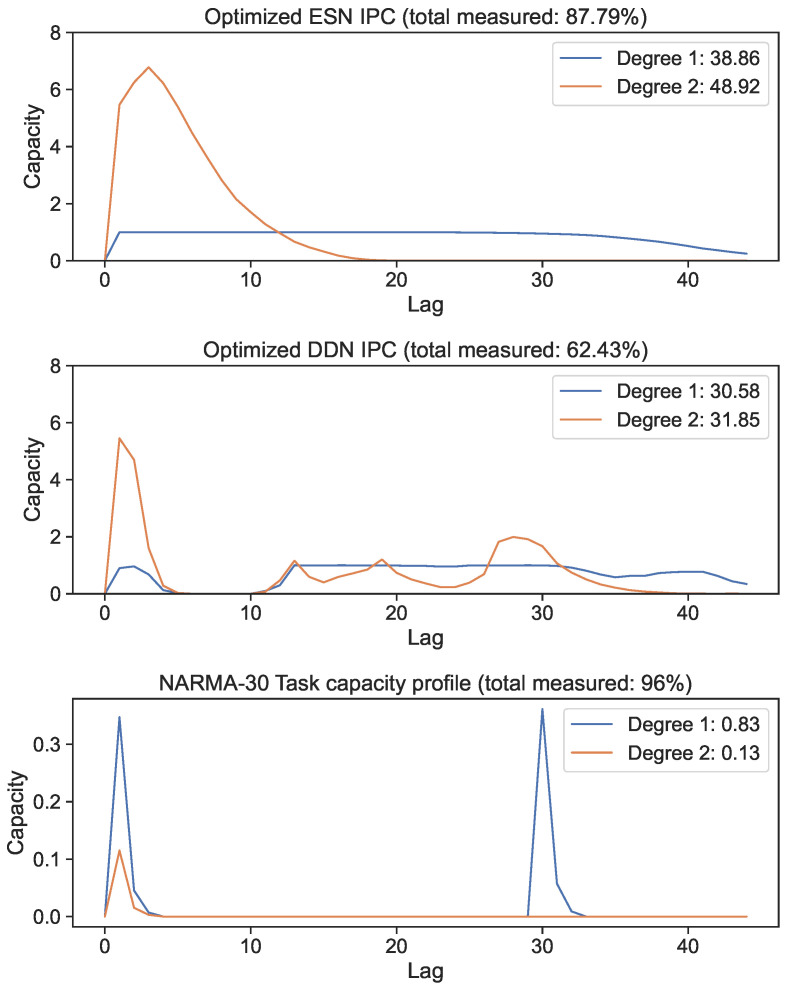
First- and second-degree IPC of networks optimized for the NARMA-30 task. Capacity profiles are the average of 10 reservoirs. The legend shows the total capacity for each degree, computed as the sum of capacities over all lags. The bottom graph shows the task capacity of both tasks, indicating task information processing requirements.

We optimized DDN and ESN hyperparameters for the NARMA-30 system approximation task using the CMA-ES. We generated 10 ESN reservoirs and 10 DDN reservoirs from the best hyperparameter candidates, with both model types comprised of 100 neurons in four clusters. The exact hyperparameter settings of the best networks are shown in Appendix A for both ESNs and DDNs. IPCs up to a degree of two were computed for each of the 10 sampled networks and averaged. In Figure 5, we show the best candidate’s IPC profiles up to a lag of 60 for the ESN and DDN models. These profiles are compared with the NARMA-30 task capacity (bottom graph). We observe that both ESNs and DDNs were able to maintain an equally high linear MC up to a lag of 30, and both had a high second-order IPC at a lag of 1. Next, we see that the total IPC measured for ESNs was higher compared to DDNs for both the linear and quadratic IPC. We observe that the task requirements were focused at a lag of one and thirty for linear memory, and at a lag of one for the second-order IPC. At first sight, this result seems contradictory to the fact that DDNs achieve a better task performance than ESNs, because the task overlap seems to be equivalent for both network types.

**Figure 6:**
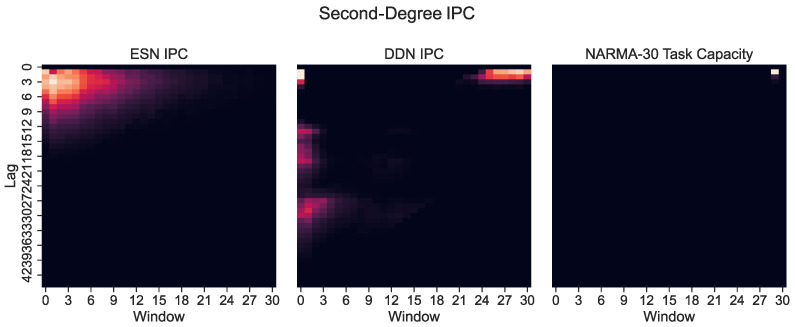
Second-degree IPC for optimized ESNs and DDNs (**left**) and DDNs (**middle**) and the NARMA-30 degree task capacity (**right**), shown as a heatmap over the lag and window size. A lighter hue indicates a higher capacity. The window size indicates the difference between the smallest and largest delay in the production of polynomials (the basis function) used to compute the capacity. We observe that the second-order task requirements are concentrated at a window size of 30.

However, only plotting the capacity against the lag ignores the IPC window size (i.e., the difference between the largest and the smallest delay in the basis function). Note that these networks were optimized for the NARMA-30 task. Therefore, the largest linear memory requirements for the system were at lag one and lag thirty. By looking at Equation (10), we see that the NARMA-30 task also requires second-order computations: multiplying the input from 30 steps ago with the input from 1 step ago. This corresponds with a window size of 29. Indeed, when we plot the IPC for ESNs and DDNs and the NARMA-30 task capacities as a heatmap over the window size and lag (Figure 6), it becomes clear that there is no second-degree task overlap at all for the ESNs, as they do not achieve non-zero capacities for such large window sizes. In accordance with findings presented by [27], we see that DDNs are able to focus the IPC in specific regions in the capacity search space. In contrast, ESNs can only achieve memory over a larger lag span by operating in a more linear regime, due to IPCs that monotonically decrease with the lag and window size. Traditionally, this is inherent to the fading memory property of ESNs. DDNs offer more tuning flexibility in terms of the IPC, allowing for a high IPC at higher degrees, higher lags, and higher window sizes compared to ESNs. Since the only difference between ESNs and DDNs is the presence of delays, we can attribute the flexibility in IPC profiles to the tuning of delays, which allows for the concentration of the IPC in task-relevant capacity regions by sacrificing the IPC in irrelevant regions. Even though we do not see a large difference between the total IPCs, the DDN IPC profiles are much better aligned with the task capacities.

**Figure 7:**
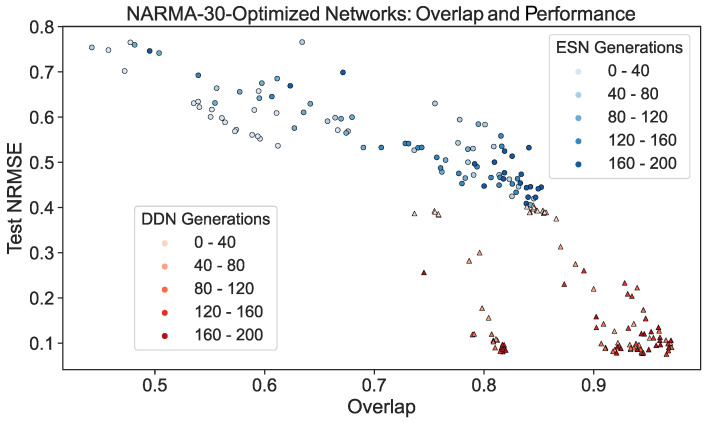
ESN and DDN NARMA-30 test performance and overlap score throughout CMA-ES evolution. Each dot represents a single instantiation of a network. The same network activity was used to measure the performance and the regional IPC, which was then used to compute the NARMA-30 overlap score. The shade of the dot indicates the CMA-ES generation from which the network was sampled. Networks were sampled from the best candidate hyperparameter set of every 10th generation, and five re-initializations were performed for each candidate. For readability, the generations were binned in five bins. Similarly to Figure 4, we see that task overlap and performance are correlated in both ESNs and DDNs. Moreover, we see that DDNs start off with a higher overlap and reach a higher overlap after hyperparameter optimization.

To further demonstrate this effect, we repeated the experiment discussed in Section 4.1. Using the validation scores from the CMA-ES optimization of ESNs and DDNs for the NARMA-30 task, we picked the best performing candidate hyperparameter set from every 10th generation. From each candidate, we sampled five networks. Each was re-trained and re-evaluated on a NARMA-30 test set. With the same network inputs and activity, the overlap score (Equation (9)) was computed. We present a scatter plot of all network instantiations throughout the evolution in Figure 7. Here, we observe that, besides having a better task performance, optimized DDNs indeed achieved a higher task overlap than ESNs, confirming that what we observe in Figure 6 is true in general, not only for the best candidates.

We summarize the NARMA-30 results for optimized and unoptimized networks in Table 1. In comparison with mixture reservoirs [2], which obtain an NRMSE of around 0.25 for NARMA-5, DDNs reach a much lower error for NARMA-30 with the same reservoir size.

### 4.3. Delayed XOR

To allow for a comparison between ESNs and DDNs throughout a range of different delays *k*, we would ideally optimize both systems for each of the 19 delays. This would result in 19 different hyperparameter sets. However, to decrease the optimization time, we instead chose to only run a single CMA-ES optimization for each model type, where a random *k* was sampled for each candidate evaluation (similarly to the approach in [14]). Because the task delay was random, and we performed multiple evaluations per candidate, the solutions with the highest fitness will be those that can perform well for most or all of the possible task delays, since over time, candidate solutions would be equally exposed to all XOR versions. As such, the optimization would ideally result in a single hyperparameter solution able to generate networks that generalize between the different task versions. This approach is sub-optimal from an application perspective, since the networks are not optimized to maximize their memory capacity at a specific lag. However, it is ideal to show the interplay between the non-linear processing power and memory, since generalizing over many different delays for such a non-linear task requires a larger short-term memory span compared to focusing on a single delay.

**Figure 8:**
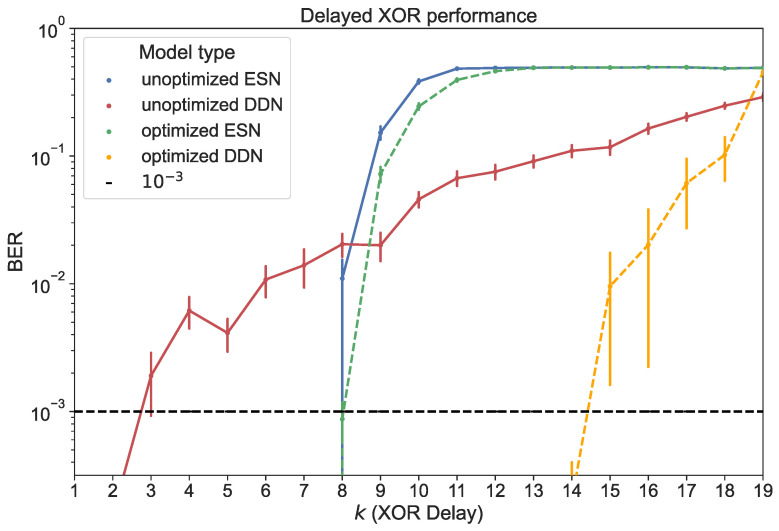
Test performance for ESNs and DDNs measured in BER, both before and after CMA-ES optimization. The horizontal axis shows the task delay used to generate each of the 19 delayed XOR test sets. BERs are averaged over 20 network samples. With a test set of $10^{4}$ samples, BERs higher than 10^−3^ are reported at a 90% confidence level [39]. The vertical error bars represent the standard error.

For both ESNs and DDNs, we used a reservoir size of 200 neurons, equally distributed in four clusters, with a (global) non-zero connectivity percentage of 25%, a weight mean of zero, a weight scaling of one, a bias mean of zero, a bias scaling of 0.5, and a leak rate of 0.99. In the case of DDNs, which required neuron location parameters, the cluster centers were spread out evenly across the diagonal of the 2D space from which the neuron locations were sampled. The cluster variances in both the *x*- and *y*-axis corresponded to 0.84 delay steps, with an x-y correlation of zero. Similarly to the findings reported in [14,17], we see that DDNs achieved a lower validation NRMSE. The best performing ESN and DDN candidates were each used to generate 20 networks. These were all trained and evaluated for all 19 different task delays, each time using a training set of 4000 samples and a test set of 10,000 samples. We report the BER for the test set in Figure 8.

We compared the task delay-dependent performance between ESNs and DDNs for optimized and unoptimized networks. Here, unoptimized networks were distributed according to the starting hyperparameters mentioned above. We note that ESNs were able to perfectly classify the delayed XOR output for lower task delays, but quickly decayed into random guessing as the task delay approached 12. Hyperparameter optimization only resulted in marginal improvements, extending the memory range of the networks to one additional task delay step.

Although unoptimized DDNs started out with a higher BER for XOR delays below nine, we observed a slower degradation of performance as the task delay was increased compared to conventional ESNs. For k≥9, unoptimized DDNs outperformed ESNs. In addition, we observed a larger effect of hyperparameter optimization for DDNs, and only a marginal effect for ESNs. Optimized DDNs maintained a BER below $10^{-3}$ up to k=14. However, we note that the use of delays seemed to increase the variability in quality between different network samples.

## 5. Discussion

In this paper, we showed that variable inter-neuron delays in recurrent neural networks improve the memory–non-linearity trade-off inherent to dynamical systems. We emphasize that the improvement of this trade-off means improving the total available usable and task-relevant information processing capacity. To demonstrate this claim, we used several approaches.

First, we showed that the random use of delays in unoptimized, randomly initialized DDNs results in a larger measured total IPC compared to ESNs. We further observed that unoptimized ESNs tend to have more non-linearity and unoptimized DDNs a longer range linear memory. However, in [1] it was previously shown that the total IPC is determined by the number of observed variables in the dynamical system (in this case, the number of neurons in the reservoir), and as such, an increase in linear memory capacity must necessarily come at the cost of non-linear computation. The measured IPC can be higher or lower depending on how we limit the capacity search space and on how many capacities are statistically insignificant. Nevertheless, we observed a better balance between memory and non-linearity in DDNs, even though the theoretical total IPC was constant, because they achieved a higher non-linearity at larger memory spans. To explain these findings, it is more relevant to only consider the portion of the IPC that is ‘useful’. The notion of the useful IPC can only be defined in the context of a task. To quantify this, we used the task capacity, reflecting the computational requirements of a task. We computed an overlap score between the task capacity profile and the reservoir IPC profile as an inner product of the capacity vectors. We see that task performance and task overlap are highly correlated metrics, further supporting the hypothesis that the improved DDN task performance is due to strong non-linearity with larger memory spans, as required by the NARMA-30 task.

However, the random introduction of delays does not reflect the improved tuning flexibility of DDNs. As such, we measured the IPC and task overlap for ESNs and DDNs optimized for the NARMA-30 task. We see that optimized DDNs achieved a better overlap score compared to ESNs. Similarly to the findings on linear memory capacity presented in [27], we see that DDN IPC profiles were not bound to monotonically decrease with delay parameters such as the lag and delay window. Instead, DDNs were flexible enough to have concentrated peaks in the IPC in specific areas, while conserving capacity in irrelevant computations. On the other hand, we consistently observed monotonically decreasing ESN capacity profiles, both for linear memory and the non-linear IPC. ESNs can only increase their capacities at any delay by increasing all previous capacities. This can be explained by the fact that, to increase their linear memory, ESNs must operate in a more linear regime. Any information passed through a non-linear activation function will decrease linear memory. Therefore, it is impossible for ESNs to achieve strong non-linearity at large lags. Although the total IPC is theoretically still the same as for equivalent DDNs, we can conclude that DDNs achieve a better balance between memory and non-linearity, not because they can increase both at the same time, but because they can have peaks in the non-linear capacity at large lags by offloading linear memory to the distributed inter-neuron delays.

Moreover, we also showed that in the case of optimized ESNs and DDNs, the task overlap is correlated with task performance. Throughout hyperparameter optimization, DDNs achieved a lower task NRMSE and higher task overlap, again indicating better tuning flexibility.

Lastly, we showed an improved performance of DDNs for the bitwise delayed XOR task. This provides another example showing a better flexibility of DDNs in producing strong non-linearities at large delays. As a result of the superior generalization power of DDN hyperparameter solutions to different delayed XOR timescales, we thus obtained a generic digital task solver. With XOR being one of the most challenging digital tasks, such a solution can be of great interest to the physical/photonic reservoir computing field.

Although it has been shown before that DDNs improve linear memory capacity at larger lags, we show for the first time that DDNs do this without reducing useful, task-specific non-linearity.

With these conclusions and previous findings, it has become clear that the use of distributed delays can be beneficial for RNNs. However, current limitations are the costly optimization procedures for finding task-relevant delay distributions. Future DDN research could focus on a better ‘genotype’ encoding of the network structure and topology, meaning that instead of simply encoding all network aspects as distributions, specific microcircuit patterns could be captured by the optimized hyperparameters. Another important future research direction is testing the added value of inter-neuron delays in real-world spatiotemporal problems, such as robot control [12], pattern generations for time series (e.g., weather forecasting, electric load forecasting [11], and financial forecasting [10]), and the enhancement of physical reservoirs with an optimized linear inter-node delay. Lastly, the effect of delay tuning on network stability and the proximity to chaotic behavior should be studied in future work.

## Figures and Tables

**Table 1:** NARMA-30 results summary for optimized and unoptimized DDNs and ESNs: means ± standard deviations. For optimized performance and overlap, means over multiple instantiations are reported. For unoptimized networks, difference scores are reported, since networks are identical, with only delays differing.

	Equivalent Nr. of Generations		Equivalent Hyperparameters
	Optimized ESN	Optimized DDN	Unoptimized ESN–Unoptimized DDN
NARMA-30 NRMSE	0.455 ± 0.070	0.087 ± 0.018	0.187 ± 0.170
NARMA-30 Task overlap	0.783 ± 0.066	0.940 ± 0.011	−0.192 ± 0.197

## Data Availability

The raw data supporting the conclusions of this article will be made available by the authors on request.

## Abbreviations

The following abbreviations are used in this manuscript:

RNN	Recurrent neural network
RC	Reservoir computing
ESN	Echo state network
LSM	Liquid state machine
BPTT	Back-propagation through time
DDN	Distance-based delay network
ADDN	Adaptive distance-based delay network
IPC	Information processing capacity
MC	Memory capacity
CMA-ES	Covariance matrix adaptation evolutionary strategy
GMM	Gaussian mixture model
NARMA	Non-linear autoregressive moving average
NRMSE	Normalized root mean squared error
XOR	Exclusive or

## Appendix A. Hyperparameter Settings

In the following tables, the optimal hyperparameter settings found by the CMA-ES evolution are presented for baseline echo state networks (ESNs) and distance-based delay networks (DDNs). Some hyperparameters are defined per cluster, whereas others are defined between each cluster pair. Location parameters are only given for DDNs, as ESNs do not contain neuron location information.

### Appendix A.1. ESN

**Table A1:** Best ESN hyperparameters per cluster. Mixture components indicate the distribution of neurons over the 4 clusters, and sum to 1.

	Cluster 1	Cluster 2	Cluster 3	Cluster 4
Mixture components	0.24	0.57	0.13	0.06
Bias weight scaling	0	0	0	0
Decay parameter	1	1	1	0.983

**Table A2:** Best ESN weight scaling per cluster-to-cluster projection. Weight scaling factors are multiplied with the uniformly distributed weights sampled between −0.5 and 0.5. Scaling factors are non-zero.

Weight Scaling	Cluster 1	Cluster 2	Cluster 3	Cluster 4	Input Neuron
Cluster 1	0.000	0.201	0.140	0.212	0.000
Cluster 2	0.494	0.572	0.834	0.061	0.244
Cluster 3	0.617	0.000	0.000	0.000	0.349
Cluster 4	0.000	0.803	0.611	0.000	0.767

**Table A3:** Best ESN connectivity per cluster-to-cluster projection. Connectivity varies between 0 and 1, and indicates percentage of non-zero weights.

Connectivity	Cluster 1	Cluster 2	Cluster 3	Cluster 4	Input Neuron
Cluster 1	0.867	1.000	0.688	0.905	0.920
Cluster 2	0.511	0.367	1.000	0.692	1.000
Cluster 3	0.850	1.000	0.860	1.000	1.000
Cluster 4	0.481	1.000	0.838	0.769	0.783

### Appendix A.2. DDN

**Table A4:** Best DDN hyperparameters per cluster. Mixture components indicate the distribution of neurons over the 4 clusters, and sum to 1. Here, one time step of delay corresponds to an inter-neuron distance of $2.4\times 10^{-4}$.

	Cluster 1	Cluster 2	Cluster 3	Cluster 4
Mixture components	0.73691	0	0.07961	0.18348
Means, X	0.00073	0.00186	0.00200	0.00064
Means, Y	0.00080	0.00231	0.00343	0.00343
Variance, X	0.00018	0.00065	0	0.00103
Variance, Y	0	0.00066	0	0
Correlation, X-Y	0.990	−0.990	−0.859	−0.218
Bias weight scaling	1.930	0.169	0.401	0.000
Decay parameter	0.564	1.000	1.000	0.183

**Table A5:** Best DDN weight scaling per cluster-to-cluster projection. Weight scaling factors are multiplied with the uniformly distributed weights sampled between −0.5 and 0.5. Scaling factors are non-zero.

Weight Scaling	Cluster 1	Cluster 2	Cluster 3	Cluster 4	Input Neuron
Cluster 1	0.000	1.200	0.000	3,038	0.000
Cluster 2	0.776	0.000	0.000	0.000	0.000
Cluster 3	0.000	0.006	0.529	0.610	0.000
Cluster 4	0.000	0.000	0.638	0.000	0.901

**Table A6:** Best DDN connectivity per cluster-to-cluster projection. Connectivity varies between 0 and 1, and indicates percentage of non-zero weights.

Connectivity	Cluster 1	Cluster 2	Cluster 3	Cluster 4	Input Neuron
Cluster 1	1.000	0.602	0.174	1.000	0.924
Cluster 2	1.000	1.000	0.458	1.000	0.998
Cluster 3	0.698	1.000	1.000	1.000	1.000
Cluster 4	1.000	1.000	0.516	1.000	0.250
